# Adherence to plant-based diet and risk of heart failure among middle-aged and older population

**DOI:** 10.3389/fnut.2026.1769535

**Published:** 2026-02-25

**Authors:** Xiao-Xin Chang, Yong-Jian Zhu, Wen-Liang Che, Yi-Ming Li

**Affiliations:** 1 Department of Cardiology, Shanghai Tenth People’s Hospital, Tongji University School of Medicine, Shanghai, China; 2 Department of Cardiology, The First Affiliated Hospital of Zhengzhou University, Zhengzhou, China; 3 Department of Cardiac Function, The Third Affiliated Hospital of Zhengzhou University, Zhengzhou, China

**Keywords:** cohort, epidemiology, heart failure, plant-based diet, risk factors

## Abstract

**Background:**

Plant-based diets have been demonstrated to be associated with lower risk of several chronic diseases. However, a comprehensive assessment of plant-based diet quality and its association with heart failure (HF) is limited. This study aimed to investigate whether healthful and unhealthful plant-based dietary patterns are associated with overall HF risk among middle-aged and older adults in the United Kingdom.

**Methods:**

We included a total of 190,092 UK Biobank participants without HF at baseline. Three plant-based diet indices were calculated using 24-h dietary recalls based on 17 food groups: the overall plant-based diet index (PDI), healthful plant-based diet index (hPDI), and unhealthful plant-based diet index (uPDI). Multivariable Cox regression models were applied to assess the association between these indices and incident HF.

**Results:**

Over a median follow-up of 13.04 years, 4,351 cases of new-onset HF were recorded. Compared to the lowest tertile, the multivariable-adjusted hazard ratios (HRs) with 95% confidence interval (CI) for HF in the highest tertile were 0.94 (0.88–1.02) for overall PDI, 0.84 (0.78–0.91) for hPDI, and 1.11 (1.03–1.19) for uPDI.

**Conclusion:**

A higher intake of healthful plant-based diets was associated with a lower risk of HF, while follow unhealthful plant-based diet was linked to a higher overall HF risk. Adhering to a high-quality diet primarily based on healthy plant-based foods may be helpful prevent HF.

## Introduction

Heart failure (HF) is a complex clinical syndrome characterized by structural and functional cardiac abnormalities that impair ventricular contraction or filling. This results in decreased cardiac output or increased intracardiac pressure, both at rest and during exercise, with primary symptoms including reduced exercise tolerance and fluid retention (1). Globally, more than 56 million people are affected by HF (2), and its prevalence has risen in part due to advances in treatment for other cardiac conditions such as myocardial infarction, valvular disease, and arrhythmias, which have extended patients’ lifespans (3). However, hospitalizations and mortality due to HF continue to increase, with its global prevalence approaching epidemic levels (4, 5). The high incidence and mortality rates, coupled with significant healthcare costs and a reduced quality of life, have made HF a substantial public health concern worldwide. HF is progressive, and once symptomatic HF develops, mortality risk can increase nearly nine-fold (6). Individuals at risk or in the early stages of HF far outnumber those with diagnosed HF, emphasizing the importance of prevention. Despite advances in treatment, clinical interventions address only a fraction of HF care, highlighting the need for preventive strategies (7).

In recent years, research has increasingly focused on dietary patterns in relation to chronic disease risk, aiming to capture the overall dietary intake and potential interactions among various food components. Plant-based diets, including the traditional Mediterranean and vegetarian diets, are typically characterized by high consumption of plant foods and minimal or no intake of animal products (8). Evidence suggests that these diets—often rich in fiber, vitamins, and bioactive compounds—may reduce the risk of chronic diseases such as cardiovascular disease by mitigating oxidative stress and inflammation and providing neuroprotective effects (9). However, not all plant-based foods are equally beneficial; for instance, foods like potatoes, refined sugars, and processed grains have been linked to a higher risk of chronic diseases. To distinguish among the quality of plant-based diets, researchers have developed three plant-based diet indices: the overall plant-based diet index (PDI), which emphasizes total plant food intake; the healthful plant-based diet index (hPDI), which highlights nutrient-dense plant foods such as whole grains, vegetables, nuts, legumes, coffee, and tea; and the unhealthful plant-based diet index (uPDI), which emphasizes less beneficial plant foods associated with increased chronic disease risk. Prior studies have found that adherence to a healthful plant-based diet that balances both beneficial and less beneficial plant-based foods, as well as animal foods, is significantly associated with a lower risk of type 2 diabetes, non-alcoholic fatty liver disease, frailty, and both all-cause and disease-specific mortality (10–13). However, there is limited evidence regarding the association between adherence to healthful plant-based dietary patterns and the risk of HF. Therefore, we conducted a large cohort study using data from the UK Biobank to evaluate the association between a healthful plant-based dietary pattern and HF risk.

## Methods

The UK Biobank is a large, prospective, population-based health study conducted worldwide that enrolled 500,000 participants aged 36–73 years between 2006 and 2010 (14, 15). Participants completed comprehensive baseline assessments at 22 central assessment centers in England, Scotland, and Wales; further details of the study protocol have been described elsewhere (16). The UK Biobank provides participants’ demographic characteristics, lifestyle, genetic information, blood samples and environmental exposure data, and tracks their health and medical records for decades afterwards. All participants provided written informed consent to participate, and ethical approval for the research involving humans was obtained from the NHS Northwest Multicenter Research Ethics Committee (no. 11/NW/0382).

We excluded participants without dietary assessment information, participants who withdrew consent during follow-up, participants with a diagnosis of HF at baseline. As a result, a total of 190,092 participants were included in the final analysis.

### Assessment of HF

HF was identified through hospital inpatient records and death registry data, based on the International Classification of Diseases, 10th Revision (ICD-10), using codes I11.0, I13.0, I13.2, I50.0, I50.1, and I50.9 (17). Data on hospital admissions extended up to October 2022 in England, August 2022 in Scotland, and May 2022 in Wales. Participants were monitored from the date of their visit to the assessment center until the first of these events: death, diagnosis of HF, or end of the follow-up period (October 2022 in England, August 2022 in Scotland, and May 2022 in Wales).

### Healthful plant-based dietary patterns

This study followed the previous research protocol and used the Oxford WebQ tool to record the average food intake over at least two 24-h periods, covering 200 common foods and 30 beverages (18). Different scores were given based on the participants’ intake of healthful plant foods, unhealthful plant foods, and animal products to construct a hPDI and an uPDI (19). The PDI consists of 17 food categories, including healthy plant-based foods (whole grains, fruits, vegetables, nuts, legumes, and vegetarian protein substitutes, as well as tea and coffee), unhealthy plant-based foods (fruit juices, refined grains, potatoes, sugary drinks, sweets and desserts), and animal-based foods (animal fats, dairy products, eggs, fish or seafood, meat, and other animal-derived foods) (20). A complete list of food groups with examples is provided in Supplementary Table S1. All of the above foods, except vegetable oils, can be used to calculate hPDI (21). The 17 food groups were assigned quintile-based scores, with healthier options receiving positive scores (Q5 = 5, Q1 = 1) and unhealthy options receiving reverse scores. The hPDI was calculated by summing the positive scores for healthy plant foods and the reverse scores for unhealthy plant and animal foods. Conversely, the uPDI was derived using the opposite scoring approach (22).

### Covariates

Covariates were selected *a priori*, mainly based on literature review (16, 23). The following variables were included: demographic characteristics—sex (male/female), age (years), education level (college/university vs. other), and employment status (employed/unemployed)—along with other potential confounders associated with HF. These comprised body mass index (BMI, kg/m^2^), physical activity (PA) (low/moderate/high), alcohol consumption (never/previous/current), smoking status (never/previous/current), and history of chronic diseases, including cardiovascular disease (yes/no) and cancer (yes/no).

### Statistical analysis

Baseline continuous variables were summarized with means and standard deviations (SD), and categorical variables were described by counts and percentages. Missing data for covariates were handled with imputation using the “mice” package in R.

To examine HF risk across tertiles of various plant-based diet indices, Cox proportional hazards models were applied to calculate hazard ratios (HRs) with 95% confidence interval (CI). The plant-based diet indices was analyzed both categorically by tertile (using the lowest tertile as reference) and continuously as per 1-SD increment. Two models were used progressively: Model 1 was unadjusted; Model 2 adjusted for age, sex, Townsend deprivation index, PA, education, employment status, smoking, alcohol consumption, BMI, CVD and cancer. A restricted cubic spline was employed to explore the dose–response relationship between continuous various plant-based diet indices and incident HF.

Subgroup analyses were conducted to assess if the association between plant-based diet indices and HF was influenced by age (≤65 or >65 years), sex (male or female), BMI (<25 or ≥25 kg/m^2^), ethnicity (White or Others), education (college/university vs. other), PA (low or moderate/high), employment status, smoking status (never or ever), alcohol consumption (never or ever). Interaction terms (e.g., potential modifier × plant-based diet index) were introduced in Model 2 for each potential modifier.

Two sensitivity analyses were conducted to test the robustness of the findings: (1) excluding participants with fewer than 2 years of follow-up, and (2) restricting the analysis to those with complete data (complete case analyses).

## Results

A total of 190,092 participants were included in the cohort, with a mean age of 56.42 ± 7.94 years, and 43.7% were female. During a mean follow-up of 13.04 years, 4,351 incident HF cases were identified. Compared with participants without HF, those who developed HF were older, more likely to be male, less physically active, had lower educational attainment, and were more frequently unemployed. They were also more likely to be current smokers and had higher Townsend deprivation index scores and BMI (Table 1). Baseline characteristics according to tertiles of hPDI, PDI, and uPDI scores are presented in Supplementary Tables S2–S4.

**Table 1:** Baseline characteristics of the participants included in this study.

Characteristics	Total (*n* = 190,092)	Incident heart failure		*p*
		No (*n* = 185,741)	Yes (*n* = 4,351)	
BMI, kg/m^2^	26.91 (4.64)	26.86 (4.61)	29.09 (5.64)	<0.001
Age, y	56.42 (7.94)	56.28 (7.93)	62.22 (6.13)	<0.001
Sex				<0.001
Male	106,938 (56.3)	105,215 (56.6)	1723 (39.6)	
Female	83,154 (43.7)	80,526 (43.4)	2,628 (60.4)	
Ethnic				0.002
White	181,036 (95.2)	176,849 (95.2)	4,187 (96.2)	
Others	9,056 (4.8)	8,892 (4.8)	164 (3.8)	
Education				<0.001
College or university	109,547 (57.6)	106,598 (57.4)	2,949 (67.8)	
Other	80,545 (42.4)	79,143 (42.6)	1,402 (32.2)	
Employment				<0.001
Yes	72,443 (38.1)	69,748 (37.6)	2,695 (61.9)	
No	117,649 (61.9)	115,993 (62.4)	1,656 (38.1)	
Physical activity				<0.001
High	34,941 (18.4)	34,020 (18.3)	921 (21.2)	
Moderate	80,215 (42.2)	78,404 (42.2)	1811 (41.6)	
Low	74,936 (39.4)	73,317 (39.5)	1,619 (37.2)	
Smoke				<0.001
Never	108,457 (57.1)	106,519 (57.3)	1938 (44.5)	
Previous	66,730 (35.1)	64,839 (34.9)	1891 (43.5)	
Current	14,905 (7.8)	14,383 (7.7)	522 (12.0)	
Drink				<0.001
Never	6,213 (3.3)	6,042 (3.3)	171 (3.9)	
Previous	5,680 (3.0)	5,459 (2.9)	221 (5.1)	
Current	178,199 (93.7)	174,240 (93.8)	3,959 (91.0)	

Values are mean ± SD, *n* (%), or median (IQR). BMI, body mass index.

**Figure 1:**
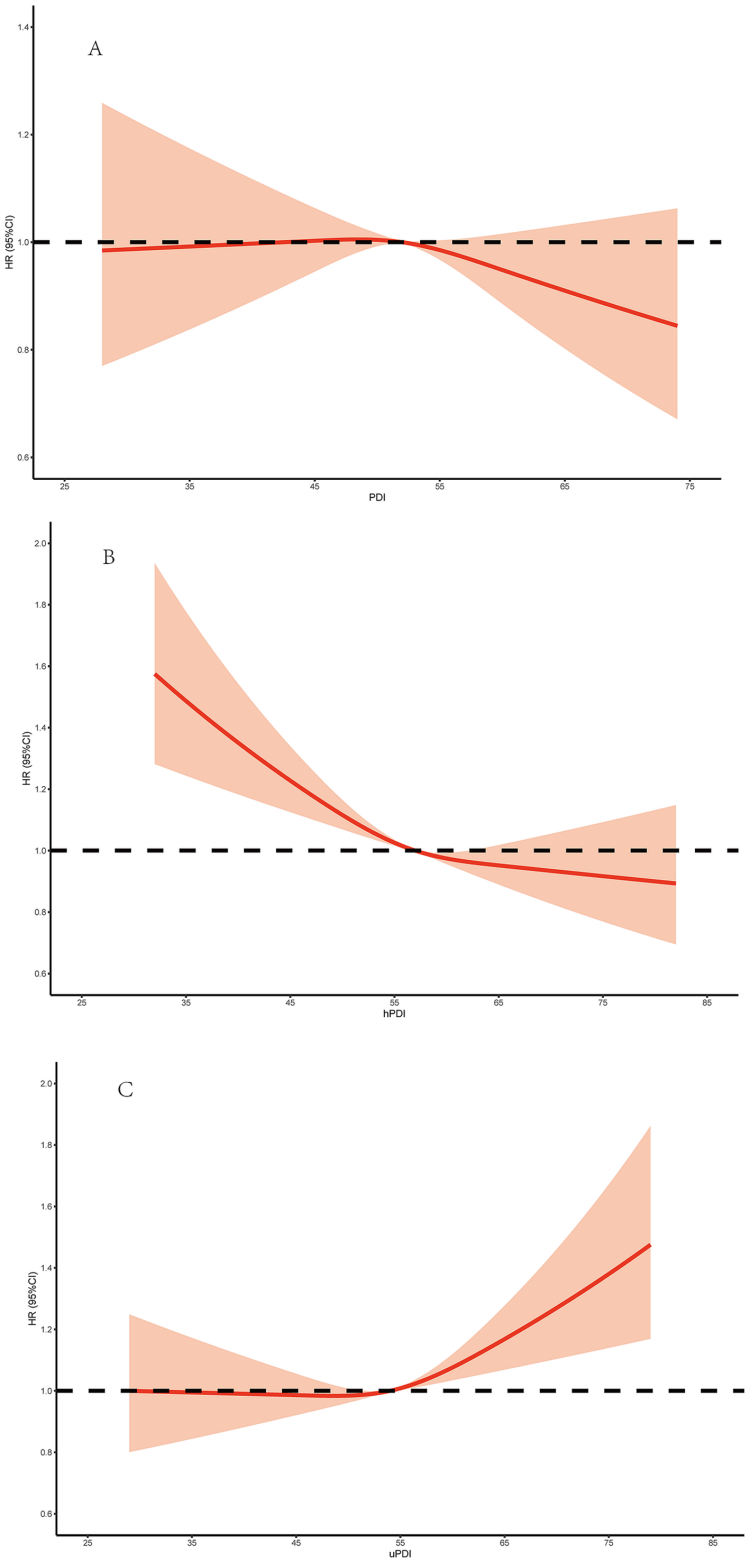
Dose–Response Relationship of PDI and hPDI, and uPDI With HF Incidence. **(A)** The dose–response relationship of the PDI with HF incidence. **(B)** The dose–response relationship of the hPDI with HF incidence. **(C)** The dose–response relationship of the uPDI with HF incidence. PDI, the overall plant-based diet index, hPDI, healthful plant-based diet index, uPDI, and unhealthful plant-based diet index, HF, heart failure.

The relationship between plant-based diet indices and incident HF is depicted in Table 2 and Figure 1. Compared to the lowest tertile, multivariable-adjusted HRs of HF in the highest tertile were 0.94 (0.88–1.02) for overall PDI, 0.84 (0.78–0.91) for hPDI, and 1.11 (1.03–1.19) for uPDI, respectively. Dose–response analysis also confirmed that the PDI risk ratio consistently remained close to 1, indicating no significant dose–response trend and no significant association with HF risk. However, hPDI showed a negative dose–response relationship with HF risk, meaning higher scores were associated with lower risk; while uPDI showed a positive dose–response relationship, with higher scores associated with higher risk (Figure 1).

**Table 2:** Associations of plant-based diet and incident heart failure.

Plant-based diet indices	Case/Pearson-years	Model^a^		Model^b^	
		HR (95% CI)	*P*	HR (95% CI)	*P*
PDI					
T1	1701/940151	Ref		Ref	
T2	1461/818712	0.98 (0.92–1.06)	0.6603	1 (0.93–1.08)	0.9457
T3	1189/719830	0.91 (0.84–0.98)	0.0126	0.94 (0.88–1.02)	0.1228
Per SD increase	/	0.96 (0.93–0.99)	0.0077	0.97 (0.94–1)	0.0715
hPDI					
T1	1857/941198	Ref			
T2	1252/727758	0.87 (0.81–0.94)	<0.001	0.88 (0.82–0.94)	<0.001
T3	1242/809737	0.78 (0.72–0.83)	<0.001	0.84 (0.78–0.91)	<0.001
Per SD increase		0.9 (0.87–0.93)	<0.001	0.91 (0.88–0.94)	<0.001
uPDI					
T1	1531/859663	Ref		Ref	
T2	1497/864791	0.97 (0.91–1.05)	0.4573	1 (0.94–1.08)	0.8912
T3	1323/754240	0.99 (0.92–1.06)	0.7371	1.11 (1.03–1.19)	0.008
Per SD increase	/	0.99 (0.96–1.02)	0.6522	1.06 (1.03–1.09)	<0.001

^a^Original model without adjusting for any variables. ^b^Adjusted for age and sex, PA, Townsend deprivation index, education, employment, smoking, drinking, BMI, CVD, and cancer. PDI, plant-based diet index; hPDI, healthy plant-based diet index; uPDI, unhealthful plant-based diet index; Ref, references.

**Figure 2:**
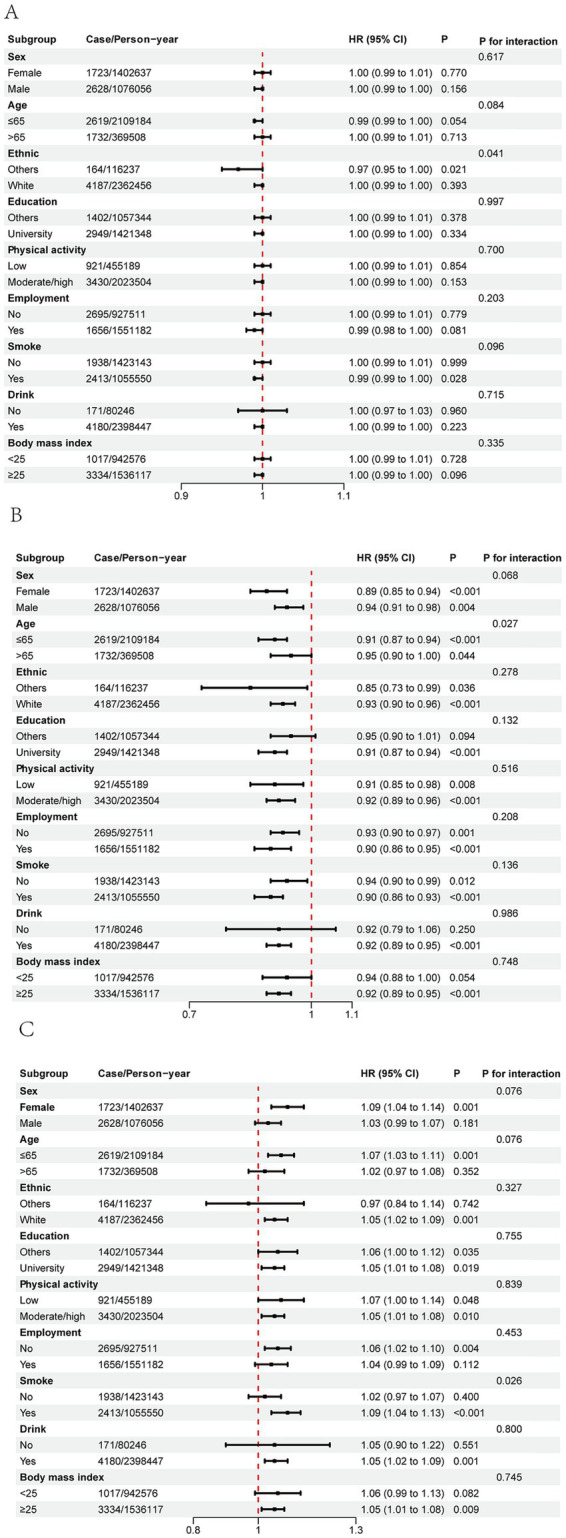
HR (95% CI) for HF comparing PDI and hPDI, and uPDI by selected characteristics. **(A)** Subgroup analysis of the associations of PDI with risk of HF. **(B)** Subgroup analysis of the associations of hPDI with risk of HF. **(C)** Subgroup analysis of the associations of uPDI with risk of HF. Forest plots showed HR and 95% CI for the highest category compared with the lowest category. All HRs and 95% CIs were estimated using Cox proportional hazard models with adjustment for sex, age, PA, education, employment, smoking, drinking, Townsend deprivation index, BMI, CVD, and cancer. BMI, body mass index; PA, physical activity.

In subgroup analyses, the overall PDI was not significantly associated with HF risk, except for a potential interaction by race. In contrast, adherence to a hPDI was consistently inversely associated with HF risk, with a stronger protective effect observed among participants aged ≤65 years. The uPDI was positively associated with HF risk overall, with a more pronounced association among current smokers. No other significant subgroup interactions were observed (Figure 2). These subgroup findings should be interpreted cautiously, as they are exploratory and hypothesis-generating (Figure 2). Sensitivity analyses confirmed the robustness of the findings regarding the associations between plant-based diet indices and incident HF (Supplementary Table S5).

## Discussion

This study found that adherence to healthful plant-based diets was associated with a lower risk of HF, whereas intake of unhealthful plant-based diets were associated with a higher risk of HF. These associations were independent of lifestyle, sociodemographic characteristics, and multiple other risk factors.

In recent years, more and more studies have begun to focus on the relationship between dietary patterns and HF. Although the guidelines have not yet recommended a specific dietary pattern, there is currently more data supporting the Dietary Approaches to Stop Hypertension (DASH) diet and the Mediterranean diet. Studies have shown that the Mediterranean diet was significantly associated with lower risk of HF, especially in women, and that the diets were associated with lower levels of specific biomarkers such as NT-proBNP, a marker associated with HF (24, 25). Mediterranean diet delays ventricular remodeling and delays the progression of HF (26). However, only a few studies have investigated the association between plant-based diets and HF.

Previous studies have shown that PDI is inversely associated with the risk of CVD (27–31). People often maintain cardiovascular health by reducing animal-based diets and increasing plant-based diets but rarely consider the quality of the plant foods they eat. In the present study, no significant association was observed between the overall PDI and incident HF. This finding may reflect the heterogeneous nature of plant-based diets, as the overall PDI captures the quantity of plant-derived food intake without distinguishing between healthful and unhealthful plant foods. Consequently, the potential cardioprotective effects of nutrient-dense plant foods may be offset by adverse effects associated with refined grains, added sugars, and other unhealthful plant-based foods, resulting in an overall null association. In contrast, the healthful and unhealthful plant-based diet indices explicitly incorporate diet quality and more accurately capture meaningful differences in dietary patterns. These results suggest that diet quality, rather than plant-based intake per se, may be more relevant to HF risk. The PDI only reflects the ratio of plant-based foods to animal-based foods consumed, without distinguishing the quality of plant-based foods. A PDI score may include both a large amount of healthy and unhealthy plant-based foods, and their combined effects are neutralized. Not all plant-based diets are beneficial for cardiovascular disease, and some types of plant-based diets may cause more harm (32). A healthy plant-based diet rich in dietary fiber, antioxidants, unsaturated fatty acids, and micronutrients may reduce the risk of HF through multiple mechanisms (33–35). Previous studies have shown that dietary fiber is associated with lower levels of inflammatory markers, while antioxidants can improve oxidative stress in the body to improve endothelial function (36). Choosing a diet rich in unsaturated fatty acids was significantly associated with improve blood lipid markers and anti-inflammatory effects (37). These ingredients all help control weight, improve insulin resistance, lower blood lipids, reduce inflammation, and promote more favorable diet-gut microbiome interactions, thereby reducing HF risk. In addition, long-term adherence to a healthful plant-based diet was associated with improve intestinal flora. Studies have confirmed that long-term healthful plant-based diet is associated with a lower relative abundance of streptococcus peptic ulcer, which is negatively correlated with high-density lipoprotein cholesterol (38–40). However, unhealthful plant-based diets are often low in fiber, poor in micronutrients, overly processed, and energy-dense, which may adversely affect the above pathways and are associated with an increased risk of overall HF. After fully adjusting for variables, participants who adhered to the plant-based dietary pattern had a lower risk of HF than those with the lowest adherence rate, while participants who consumed more unhealthy plant-based diets had a higher risk of HF than those who consumed less unhealthy plant-based diets. Dose–response analysis revealed a consistent monotonic increase in HF risk with higher levels of uPDI, but a lower risk for higher hPDI. This finding suggests that patients with risk factors for HF need to consider whether their plant-based diet is healthy.

In this study, we also conducted a series of sensitivity analyses to ensure the robustness of our findings. Furthermore, to consider the diversity of diets, we calculated dietary scores instead of single nutrients. This is consistent with 2021 Dietary Guidance to Improve Cardiovascular Health, which recommend a more overall healthy eating pattern rather than individual nutrients (41). To assess the robustness of our findings, we conducted extensive sensitivity analyses addressing potential residual confounding and reverse causation. The consistent, dose-dependent relationships observed, where higher hPDI was linearly associated with lower HF risk, and higher uPDI with elevated risk, suggesting that the associations are unlikely to be fully explained by unmeasured confounders, as residual confounding typically does not produce such graded, monotonic response patterns. Furthermore, the stability of these associations across nearly all subgroups in stratified analyses, the observed consistency, and their generalizability, and reduces the likelihood that they are driven by confounding specific to certain populations. To mitigate reverse causality, we performed analyses excluding participants with early-onset HF or major dietary changes within the first two years of follow-up. The persistence of significant associations in these sensitivity analyses suggests that pre-existing undiagnosed disease is unlikely to be the primary driver of our results.

Although this study is a large sample with comprehensive data on diet, risk factors, and confounders of HF risk, it has several limitations. First, the dietary intake was self-reported via a 24-h online questionnaire, which may not be representative of long-term dietary habits and may cause measurement errors. To improve reliability, participants with only a single recall were excluded, and the mean intake was calculated for those with two or more assessments. In addition, residual measurement error remains possible, but it is likely non-differential with respect to incident HF and would tend to attenuate, rather than exaggerate, the observed associations. Second, we did not differentiate between HF subtypes (e.g., HF with preserved vs. reduced ejection fraction) in our analysis. Therefore, the observed associations pertain to overall incident HF, and it remains unclear whether similar relationships exist for specific HF phenotypes. Future studies with detailed HF subtype data are warranted to explore these associations further. Third, even though we have adjusted for a wide range of covariates, residual or unknown confounding could not be ruled out because of the observational study design. Forth, although the prospective design reduces the likelihood of reverse causation, it remains possible that subclinical or undiagnosed conditions at baseline influenced dietary patterns. Sensitivity analyses excluding participants who developed new-onset HF within 2 year of completing the last 24-h dietary recall produced similar results, supporting the robustness of our findings. Nevertheless, these limitations should be considered when interpreting the results. Finally, subgroup analyses were conducted to explore potential effect modification by sex, age, and other baseline characteristics. These analyses were not prespecified as primary hypotheses and should therefore be considered exploratory. Observed differences across subgroups are hypothesis-generating and may help guide future research, but they should be interpreted with caution and cannot be considered confirmatory.

## Conclusion

Our research found that adhering to a healthy plant-based dietary pattern inversely associated with the incidence of HF, whereas indicators of an unhealthy plant-based diet were positively associated with HF risk. These findings suggest that a healthy plant-based diet may represent a higher-quality dietary pattern in relation to HF risk. While causality cannot be inferred, adherence to an overall high-quality dietary pattern may be relevant from a public health perspective. Our results are consistent with existing dietary guidelines that emphasize greater intake of healthy plant-based foods and reduced consumption of unhealthy plant-based foods in populations at risk of HF.

## Data Availability

The datasets presented in this study can be found in online repositories. The names of the repository/repositories and accession number(s) can be found in the article/Supplementary material.
