# Supplementary material for: Adherence to plant-based diet and risk of heart failure among middle-aged and older population
Source: Front Nutr. 2026 Feb 25;13:1769535. doi: 10.3389/fnut.2026.1769535 (PMC12975577; doi:10.3389/fnut.2026.1769535)
Supplement: Supplementary file 1 [file Table_1.docx]

**Table S1 Examples of Food Items Constituting the 17 Food Groups**

| Food Groups | hPDI | Items |
| --- | --- | --- |
| Healthy Plant-based Foods | | |
| Whole grains | ✓ | Breakfast cereals, Muesli (whole-grain mixed cereal without added sugar), Bran flakes, Whole meal bread, Granary/Multi-grain bread, Whole meal pita, Rye bread, Brown rice, Whole-wheat pasta, Quinoa, Barley, Millet, Buckwheat, Bulgur wheat, Oatcakes, Whole meal biscuits. |
| Fruits | ✓ | Apples, pears, bananas. citrus fruits, berries, stone fruits, pineapple, mango, melons (cantaloupe/watermelon), kiwi, grapes, dried fruits, canned and stewed fruits. |
| Vegetables | ✓ | Broccoli, cauliflower, cabbage, Brussels sprouts, spinach, kale, beet greens, lettuce, carrots, beetroot, onions, garlic, scallions, tomatoes (and their juice), cucumbers, bell peppers/sweet peppers, eggplant, zucchini, and various edible mushrooms, other vegetables, mixed vegetables |
| Nuts | ✓ | Walnuts, almonds, cashews, pistachios, hazelnuts, Brazil nuts, macadamia nuts, peanuts. |
| Legumes and vegetarian protein alternatives | ✓ | chickpeas, lentils, kidney beans, black beans, pinto beans, fava beans, peas, green beans/string beans, edamame, soybeans, tofu, soy mince. |
| Tea and coffee | ✓ | Filter coffee, Espresso, decaffeinated coffee, instant coffee, standard tea / Black tea, green tea, Herbal/Fruit tea, Oolong tea, white tea. |
| Vegetable oils | ✓ | Olive oil, rapeseed oil, sunflower oil. |
| Unhealthy Plant-based Foods | | |
| Refined grains | × | White bread, white rice, regular pasta, white noodles, couscous, cornflakes, rice crispies, sweet biscuits, cake, scones, sausage roll pastry, pizza base. |
| Potatoes (including fried) | × | Boiled potatoes, baked potatoes, mashed potatoes, French fries, potato chips. |
| Sugar-sweetened beverages | × | Sweetened soda, energy drinks. |
| Fruit juices | × | Fruit smoothie, other fruit/vegetable juice. |
| Sweets and desserts | × | Chocolate, hard candy, soft candy, toffee, mints, jellybeans, jam, honey, syrup, white sugar, brown sugar, sponge cake, chocolate cake, fruitcake, cupcakes, muffins, donuts, pies, tarts, ice cream, custard, mousse, sweet biscuits, puddings. |
| Animal-based Foods | | |
| Dairy products | × | Milk, cheese, yogurt, cream, butter. |
| Eggs | × | Eggs (boiled, fried, scrambled, etc.) |
| Meat | × | Beef, steak, beef patties, lamb, lamb chops, pork, pork tenderloin, bacon, ham, sausage, cured sausage, salami, luncheon meat, cured meats, chicken, turkey, duck, chicken wings, chicken legs, chicken nuggets, meat pies, meat pastries, meat sauce, meatballs, offal, liver, kidneys. |
| Fish and seafood | × | White fish, cod, haddock, flounder, sole, sea bass, oily fish, salmon, mackerel, herring, trout, sardines, fresh tuna, canned tuna, shrimp, prawns, lobster, crab, mussels, scallops, oysters, squid, octopus, seafood sauce, fish sauce, mixed seafood platter. |
| Animal fats and other animal-based items | × | Butter, lard, dripping, beef tallow, cream, sour cream, double cream, spray cream, spreads containing animal ingredients, gravy, meat sauces, fish sauce, shrimp paste, sauces containing anchovies, desserts containing gelatin, pudding. |

hPDI = healthy plant-based diet index

**Table S2 Baseline characteristics of the study participants by PDI**

| **Characteristics** | **Total (n=190,092)** | **PDI** | | |
| --- | --- | --- | --- | --- |
|  |  | **T1(n=72264)** | **T2(n=62760)** | **T3(n=55068)** |
| **BMI, kg/m^2^** | 26.91 (4.64) | 27.21 (4.70) | 26.81 (4.61) | 26.63 (4.58) |
| **Age, y** | 56.42 (7.94) | 56.18 (7.95) | 56.57 (7.92) | 56.56 (7.95) |
| **Sex** |  |  |  |  |
| Male | 106938 (56.3) | 39298 (54.4) | 35990 (57.3) | 31650 (57.5) |
| Female | 83154 (43.7) | 32966 (45.6) | 26770 (42.7) | 23418 (42.5) |
| **Ethnic** |  |  |  |  |
| White | 181036 (95.2) | 68263 (94.5) | 60021 (95.6) | 52752 (95.8) |
| Others | 9056 (4.8) | 4001 (5.5) | 2739 (4.4) | 2316 (4.2) |
| **Education** |  |  |  |  |
| College or university | 109547 (57.6) | 43148 (59.7) | 35915 (57.2) | 30484 (55.4) |
| Other | 80545 (42.4) | 29116 (40.3) | 26845 (42.8) | 24584 (44.6) |
| **Employment** |  |  |  |  |
| Yes | 72443 (38.1) | 26390 (36.5) | 24314 (38.7) | 21739 (39.5) |
| No | 117649 (61.9) | 45874 (63.5) | 38446 (61.3) | 33329 (60.5) |
| **Physical activity** |  |  |  |  |
| High | 34941 (18.4) | 14543 (20.1) | 11407 (18.2) | 8991 (16.3) |
| Moderate | 80215 (42.2) | 30356 (42.0) | 26767 (42.6) | 23092 (41.9) |
| Low | 74936 (39.4) | 27365 (37.9) | 24586 (39.2) | 22985 (41.7) |
| **Smoke** |  |  |  |  |
| Never | 108457 (57.1) | 39391 (54.5) | 36365 (57.9) | 32701 (59.4) |
| Previous | 66730 (35.1) | 26136 (36.2) | 21760 (34.7) | 18834 (34.2) |
| Current | 14905 (7.8) | 6737 (9.3) | 4635 (7.4) | 3533 (6.4) |
| **Drink** |  |  |  |  |
| Never | 6213 (3.3) | 2200 (3.0) | 1980 (3.2) | 2033 (3.7) |
| Previous | 5680 (3.0) | 2050 (2.8) | 1823 (2.9) | 1807 (3.3) |
| Current | 178199 (93.7) | 68014 (94.1) | 58957 (93.9) | 51228 (93.0) |

Values are mean ± SD, n (%), or median (IQR).

BMI = body mass index

PDI = plant-based diet index

**Table S3 Baseline characteristics of the study participants by hPDI**

| **Characteristics** | **Total (n=190,092)** | **hPDI** | | |
| --- | --- | --- | --- | --- |
|  |  | **T1(n=72364)** | **T2(n=55795)** | **T3(n=61933)** |
| **BMI, kg/m^2^** | 26.91 (4.64) | 27.55 (4.82) | 26.81 (4.54) | 26.26 (4.42) |
| **Age, y** | 56.42 (7.94) | 55.56 (8.19) | 56.79 (7.86) | 57.08 (7.62) |
| **Sex** |  |  |  |  |
| Male | 106938 (56.3) | 34123 (47.2) | 32106 (57.5) | 40709 (65.7) |
| Female | 83154 (43.7) | 38241 (52.8) | 23689 (42.5) | 21224 (34.3) |
| **Ethnic** |  |  |  |  |
| White | 181036 (95.2) | 68949 (95.3) | 53272 (95.5) | 58815 (95.0) |
| Others | 9056 (4.8) | 3415 (4.7) | 2523 (4.5) | 3118 (5.0) |
| **Education** |  |  |  |  |
| College or university | 109547 (57.6) | 44732 (61.8) | 32080 (57.5) | 32735 (52.9) |
| Other | 80545 (42.4) | 27632 (38.2) | 23715 (42.5) | 29198 (47.1) |
| **Employment** |  |  |  |  |
| Yes | 72443 (38.1) | 25963 (35.9) | 21898 (39.2) | 24582 (39.7) |
| No | 117649 (61.9) | 46401 (64.1) | 33897 (60.8) | 37351 (60.3) |
| **Physical activity** |  |  |  |  |
| High | 34941 (18.4) | 14930 (20.6) | 10210 (18.3) | 9801 (15.8) |
| Moderate | 80215 (42.2) | 30615 (42.3) | 23665 (42.4) | 25935 (41.9) |
| Low | 74936 (39.4) | 26819 (37.1) | 21920 (39.3) | 26197 (42.3) |
| **Smoke** |  |  |  |  |
| Never | 108457 (57.1) | 40971 (56.6) | 32060 (57.5) | 35426 (57.2) |
| Previous | 66730 (35.1) | 24617 (34.0) | 19518 (35.0) | 22595 (36.5) |
| Current | 14905 (7.8) | 6776 (9.4) | 4217 (7.6) | 3912 (6.3) |
| **Drink** |  |  |  |  |
| Never | 6213 (3.3) | 2383 (3.3) | 1714 (3.1) | 2116 (3.4) |
| Previous | 5680 (3.0) | 2103 (2.9) | 1576 (2.8) | 2001 (3.2) |
| Current | 178199 (93.7) | 67878 (93.8) | 52505 (94.1) | 57816 (93.4) |

Values are mean ± SD, n (%), or median (IQR).

BMI = body mass index

hPDI = healthy plant-based diet index

**Table S4 Baseline characteristics of the study participants by uPDI**

| **Characteristics** | **Total (n=190,092)** | **uPDI** | | |
| --- | --- | --- | --- | --- |
|  |  | **T1(n=65816)** | **T2(n=66355)** | **T3(n=57921)** |
| **BMI, kg/m^2^** | 26.91 (4.64) | 26.62 (4.55) | 26.83 (4.57) | 27.35 (4.80) |
| **Age, y** | 56.42 (7.94) | 57.65 (7.55) | 56.64 (7.87) | 54.76 (8.17) |
| **Sex** |  |  |  |  |
| Male | 106938 (56.3) | 39316 (59.7) | 37708 (56.8) | 29914 (51.6) |
| Female | 83154 (43.7) | 26500 (40.3) | 28647 (43.2) | 28007 (48.4) |
| **Ethnic** |  |  |  |  |
| White | 181036 (95.2) | 63370 (96.3) | 63306 (95.4) | 54360 (93.9) |
| Others | 9056 (4.8) | 2446 (3.7) | 3049 (4.6) | 3561 (6.1) |
| **Education** |  |  |  |  |
| College or university | 109547 (57.6) | 35756 (54.3) | 38020 (57.3) | 35771 (61.8) |
| Other | 80545 (42.4) | 30060 (45.7) | 28335 (42.7) | 22150 (38.2) |
| **Employment** |  |  |  |  |
| Yes | 72443 (38.1) | 27669 (42.0) | 25612 (38.6) | 19162 (33.1) |
| No | 117649 (61.9) | 38147 (58.0) | 40743 (61.4) | 38759 (66.9) |
| **Physical activity** |  |  |  |  |
| High | 34941 (18.4) | 10489 (15.9) | 12145 (18.3) | 12307 (21.2) |
| Moderate | 80215 (42.2) | 27569 (41.9) | 28210 (42.5) | 24436 (42.2) |
| Low | 74936 (39.4) | 27758 (42.2) | 26000 (39.2) | 21178 (36.6) |
| **Smoke** |  |  |  |  |
| Never | 108457 (57.1) | 37076 (56.3) | 38011 (57.3) | 33370 (57.6) |
| Previous | 66730 (35.1) | 24506 (37.2) | 23344 (35.2) | 18880 (32.6) |
| Current | 14905 (7.8) | 4234 (6.4) | 5000 (7.5) | 5671 (9.8) |
| **Drink** |  |  |  |  |
| Never | 6213 (3.3) | 1861 (2.8) | 2078 (3.1) | 2274 (3.9) |
| Previous | 5680 (3.0) | 1913 (2.9) | 1866 (2.8) | 1901 (3.3) |
| Current | 178199 (93.7) | 62042 (94.3) | 62411 (94.1) | 53746 (92.8) |

Values are mean ± SD, n (%), or median (IQR).

BMI = body mass index

uPDI = unhealthful plant-based diet index

**Table S5 Association between biological aging and risk of heart failure in sensitivity analysis***

|  | **PDI** | |  | **hPDI** | |  | **uPDI** | |
| --- | --- | --- | --- | --- | --- | --- | --- | --- |
|  | **HR(95% CI)** | **P** |  | **HR(95% CI)** | **P** |  | **HR(95% CI)** | **P** |
| **Excluding participant followed up less than 2 years** | | |  |  | |  |  |  |
| Q1 | Ref |  |  | Ref |  |  | Ref |  |
| Q2 | 1(0.93 - 1.08) | 0.9642 |  | 0.87(0.81 - 0.94) | <0.001 |  | 1(0.93 - 1.08) | 0.9064 |
| Q3 | 0.94(0.87 - 1.01) | 0.1148 |  | 0.82(0.76 - 0.88) | <0.001 |  | 1.12(1.04 - 1.21) | 0.003 |
| Per SD increase | 0.98(0.95 - 1.01) | 0.1144 |  | 0.91(0.88 - 0.94) | <0.001 |  | 1.06(1.02 - 1.09) | <0.001 |
| **Restricting to participant with complete information of covariates** | | |  |  |  |  |  |  |
| Q1 | Ref |  |  | Ref |  |  | Ref |  |
| Q2 | 1.01(0.94 - 1.07) | 0.858 |  | 0.89(0.83 - 0.95) | 0.001 |  | 0.99(0.93 - 1.06) | 0.854 |
| Q3 | 1.02(0.95 - 1.09) | 0.651 |  | 0.91(0.85 - 0.97) | 0.006 |  | 1.08(1.01 - 1.15) | 0.035 |
| Per SD increase | 1.01(0.98 - 1.03) | 0.664 |  | 0.95(0.93 - 0.98) | 0.001 |  | 1.04(1.01 - 1.07) | 0.005 |

*adjusted for age and sex, PA, townsend deprivation index, education, employment, smoking, drinking, BMI, CVD, and cancer
